# Correlation of Grades of Non-alcoholic Fatty Liver on Ultrasound With Blood Parameters

**DOI:** 10.7759/cureus.53075

**Published:** 2024-01-27

**Authors:** Uffan Zafar, Muhammad Nadeem Ahmad, Naila Nadeem, Mallick Muhammad Zohaib Uddin, Burhan Zafar, Shazia Baig, Fariha Zafar, Hafsa Pervez, Saba Akram

**Affiliations:** 1 Radiology, Aga Khan University Hospital, Karachi, PAK; 2 Epidemiology and Public Health, Quaid-e-Azam Medical College, Bahawalpur, PAK; 3 Internal Medicine, Dow University of Health Sciences, Civil Hospital Karachi, Karachi, PAK; 4 Pathology and Laboratory Medicine, Aga Khan University Hospital, Karachi, PAK

**Keywords:** non-alcoholic fatty, correlation, steatosis, steatohepatitis, ultrasound, fatty liver, nafld

## Abstract

Introduction

Non-alcoholic fatty liver disease (NAFLD) is the most prevalent liver condition worldwide. NAFLD has been associated with metabolic syndrome and its symptoms, such as type 2 diabetes, hypertension, dyslipidemia, and obesity. Ultrasound is widely used to grade hepatic steatosis, being the most cost-effective, non-invasive, and readily available modality without radiation exposure. The study aimed to assess the correlation of NAFLD grade as seen on ultrasound with blood parameters in a Pakistani population.

Materials and methods

The included patients were those who were diagnosed with fatty liver disease on ultrasound and whose laboratory tests were available within two weeks of the ultrasound. Two seasoned radiologists rated the severity of NAFLD after looking over ultrasound scans. Consecutive sampling technique was used to minimize selection bias. The degree and direction of the linear relationship between the NAFLD grade and each biochemical parameter were measured using the Pearson correlation coefficient.

Results

There were 207 patients in all who had been identified with NAFLD on ultrasound, the majority of whom had grade II NAFLD and were in their sixth decade of life. According to Pearson's analysis, the grade of NAFLD had larger positive associations with triglycerides, total cholesterol, low-density lipoprotein, and fasting blood sugar. High density lipoprotein and C-reactive protein were found to have a negative correlation with the grade of NAFLD.

Conclusion

The findings of the study highlight the correlation between NAFLD grade on ultrasonography and specific blood parameters, implying that managing these biochemical indicators may help to improve hepatic steatosis.

## Introduction

Non-alcoholic fatty liver disease (NAFLD) is the most prevalent liver condition and may affect up to 25% of people worldwide [1]. NAFLD may progress and lead to chronic liver disease (CLD) and hepatocellular carcinoma (HCC) [2]. NAFLD has been linked to metabolic syndrome and its features, including hypertension, dyslipidemia, type 2 diabetes mellitus, and obesity [3]. A liver biopsy is still considered the gold standard for the diagnosis of NAFLD [4,5]. However, ultrasound is widely used to grade hepatic steatosis, being the most cost-effective, non-invasive, and readily available modality without radiation exposure [6-8].

Several researchers have evaluated the correlation of this grading of hepatic steatosis on ultrasound with the parameters thought to be associated with NAFLD. However, the results have been inconsistent [9,10]. Moreover, these studies were mostly performed in the Western world where there is greater consumption of alcohol along with binge drinking, contributing to a large burden of fatty liver disease and CLD [11]. This occasional drinking may affect the associations of NAFLD in those populations. However, due to cultural and religious restrictions on alcohol in a country like Pakistan, fatty liver results mostly from NAFLD. The majority of the population does not consume alcohol throughout their lives. As a result, there is a unique opportunity in such a population to examine the relationships linked solely to NAFLD without the interference of alcohol-related factors. In a study conducted in Pakistan, the prevalence of NAFLD was reported to be 15.3% [12].

The study aimed to assess the correlation of NAFLD grade on ultrasound with blood parameters in a Pakistani population. This correlation will help clinicians monitor and control the parameters associated with the progression of the disease.

## Materials and methods

A cross-sectional study was conducted in the Radiology Department of Aga Khan University Hospital, Karachi, between March 15 and April 15, 2023, to evaluate the relationship between blood parameters and the ultrasound grading of NAFLD. The study was approved by the Ethical Review Committee of Aga Khan University Hospital Karachi.

The study included patients diagnosed with fatty liver disease on ultrasound in our department who had specific laboratory tests available within two weeks of the ultrasound. These laboratory tests included alanine aminotransferase (ALT), aspartate aminotransferase (AST), total cholesterol (TC), triglycerides (TG), high-density lipoprotein cholesterol (HDL-C), low-density lipoprotein cholesterol (LDL-C), C-reactive protein (CRP) and fasting blood sugar (FBS). Patients with a history of alcohol consumption, hepatitis, hepatotoxic drug intake, other CLD risk factors, or those with incomplete data were excluded.

Two qualified radiologists reviewed ultrasound images and graded the severity of NAFLD using the scoring system explained by Saadeh et al. [6]. On grayscale ultrasonography images, the severity of hepatic steatosis was graded using the following scales:

Grade 0: normal liver echogenicity.

Grade I: mild increase in liver parenchymal echogenicity with normal echogenicity of the portal vein wall and diaphragm.

Grade II: moderate increase in liver parenchymal echogenicity with reduced echogenicity of the portal vein wall and diaphragm.

Grade III: severe increase in liver parenchymal echogenicity with almost no visualization of the portal vein wall and diaphragm.

The data obtained included laboratory tests from the MyPatients (electronic medical record of Aga Khan University Hospital) and ultrasound examinations from the Picture Archiving and Communication System (PACS). The ultrasound examinations were performed by experienced radiologists using a standardized protocol and equipment.

Consecutive sampling technique was used to minimize selection bias. The researchers tried to reduce measurement bias by quantifying grading from Grade I to Grade III. Descriptive statistics were used to summarize the characteristics of the participants and the distribution of NAFLD grades. The Pearson correlation coefficient was used to determine the strength and direction of the linear relationship between NAFLD grade and each biochemical marker. A p-value of less than 0.05 was selected to be statistically significant. Data analysis was conducted using IBM Statistical Package for the Social Sciences (SPSS) Statistics for Windows, Version 21 (Released 2012; IBM Corp., Armonk, NY). 

## Results

**Figure 1:**
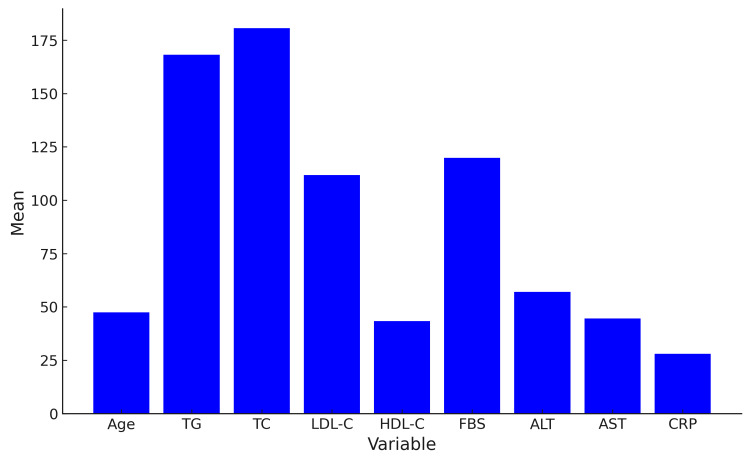
Characteristics of study participants. TG: triglycerides; TC: total cholesterol; LDL-C: low density lipoprotein cholesterol; HDL-C: high density lipoprotein cholesterol; FBS: fasting blood sugar; ALT: alanine transaminase; AST: aspartate transaminase; CRP: C-reactive protein

A total of 207 patients diagnosed with NAFLD on ultrasound were included in the study. Of the patients, 63.45% were male and 36.55% were female, with a mean age of 47.92 (SD 12.59). Grade II NAFLD was the most prevalent (42%). Figure 1 displays the characteristics of the study participants.

**Figure 2:**
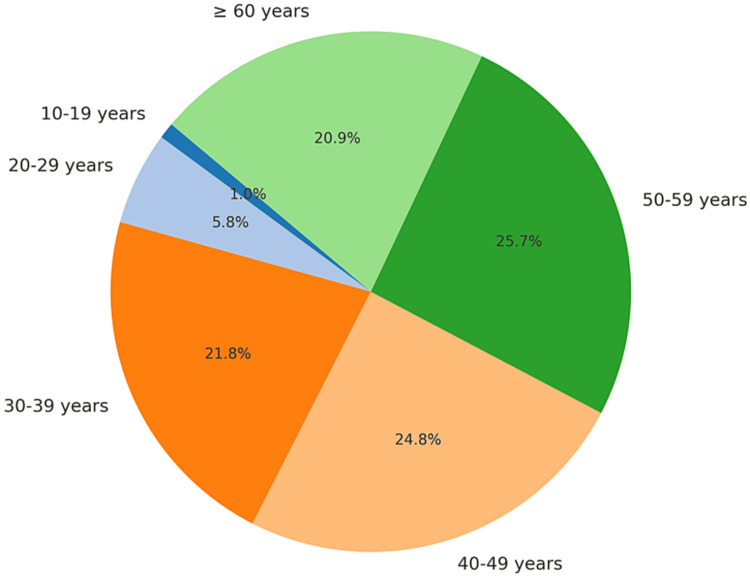
Distribution of age groups in the study participants.

The majority of the patients were beyond the age of 40 years. Only 1% of the patients were under the age of 20 years. The remaining patients in the study ranged in age from 20 to 40 years (Figure 2).

**Figure 3:**
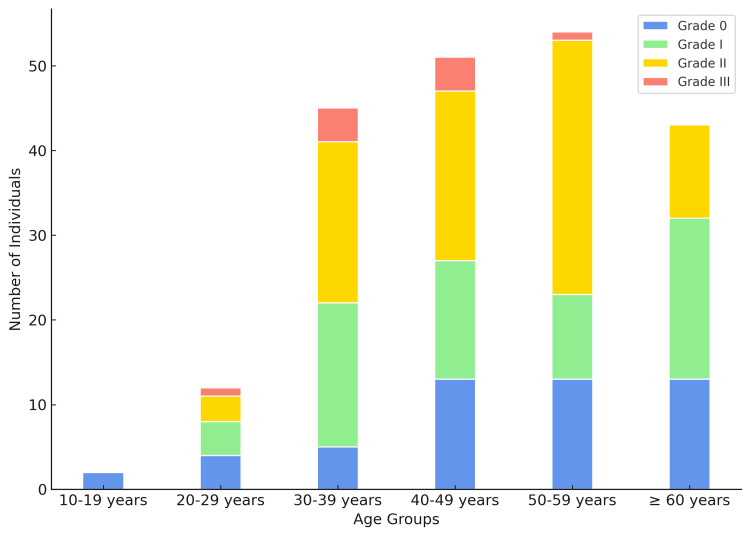
Distribution of grades across each age group.

Grade III NAFLD cases were most prevalent in the fifth decade of life, whereas Grade II cases predominated in the sixth decade. In the second decade, there were no cases of NAFLD (Figure 3).

**Figure 4:**
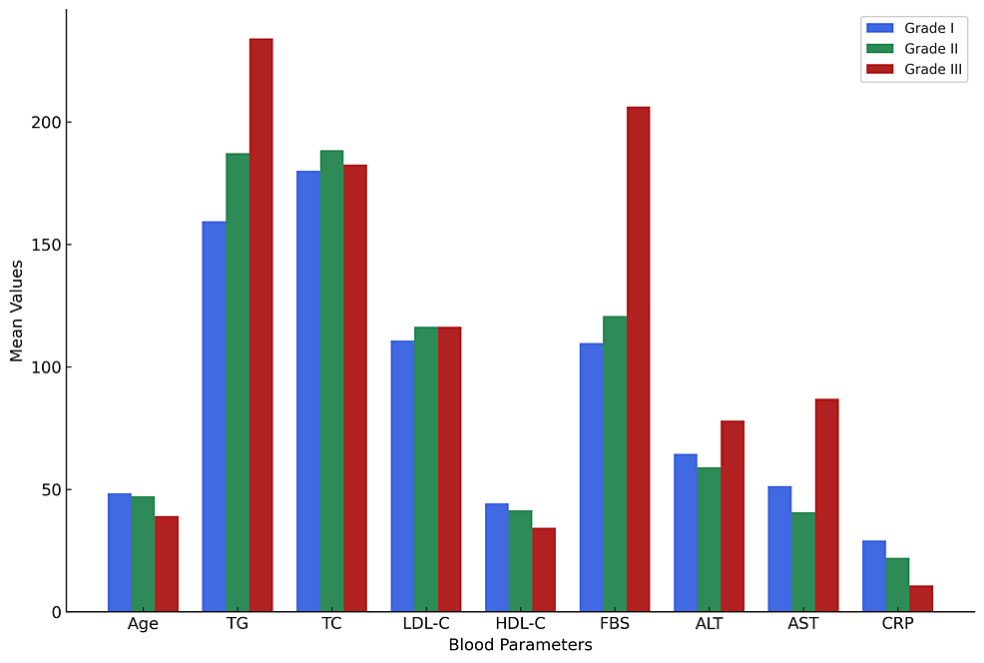
Mean values of selected blood parameters across NAFLD grades. TG: triglycerides; TC: total cholesterol; LDL-C: low density lipoprotein cholesterol; HDL-C: high density lipoprotein cholesterol; FBS: fasting blood sugar; ALT: alanine transaminase; AST: aspartate transaminase; CRP: C-reactive protein

TG, LDL-C and FBS showed direct correlation with the grades of NAFLD, i.e., the higher the grade, the higher were the values of these blood parameters. However, there was an inverse relationship between NAFLD grades and HDL-C and CRP (Figure 4).

**Figure 5:**
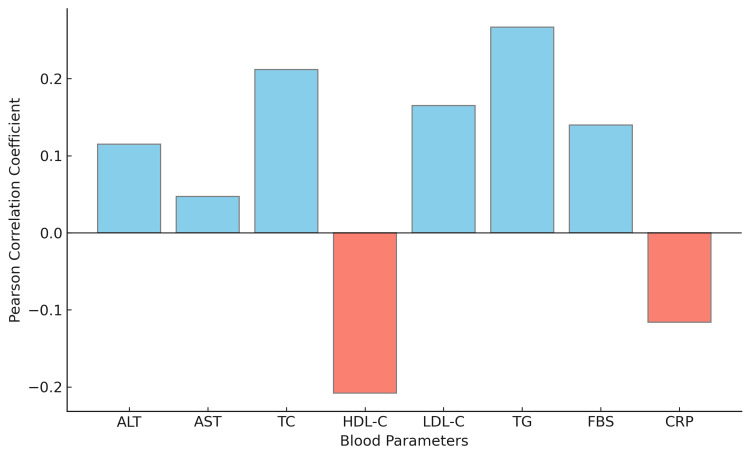
Pearson correlation of blood parameters with NAFLD grades on ultrasound. ALT: alanine transaminase; AST: aspartate transaminase; TC: total cholesterol; HDL-C: high density lipoprotein cholesterol; LDL-C: low density lipoprotein cholesterol; TG: triglycerides; FBS: fasting blood sugar; CRP: C-reactive protein.

Pearson correlation showed TG having the highest positive correlation with the grade of NAFLD, followed by TC, LDL-C and FBS (Figure 5). This suggests that the higher levels of these parameters might be associated with a higher grade of NAFLD. ALT and AST showed relatively lower correlations with the grade of NAFLD. HDL-C and CRP showed negative correlations with the grade of NAFLD, suggesting that higher levels of these parameters might be associated with a lower grade of NAFLD (Figure 5).

Statistically significant correlations with NAFLD grades were observed for TC, HDL-C, TG, and FBS. In contrast, ALT, AST, LDL-C, and CRP did not show statistically significant correlations (Table 1).

**Table 1:** Correlation analysis between NAFLD grades and blood parameters with p-values TG: triglycerides; TC: total cholesterol; LDL-C: low density lipoprotein cholesterol; HDL-C: high density lipoprotein cholesterol; FBS: fasting blood sugar; ALT: alanine transaminase; AST: aspartate transaminase; CRP: C-reactive protein; NAFLD: Non-alcoholic fatty liver disease

Blood Parameters	Pearson Correlation	P-value
ALT	0.12	0.072
AST	0.05	0.127
TC	0.21	0.032
HDL-C	-0.21	0.005
LDL-C	0.17	0.098
TG	0.27	0.001
FBS	0.14	0.001
CRP	-0.116	0.294

## Discussion

The findings of the present study add to our knowledge of NAFLD and its therapeutic consequences by shedding light on the link between the severity of NAFLD and physiological markers. The findings revealed that the grade of steatosis on ultrasound showed a correlation with the lipid profile, highlighting the potential utility of these parameters as indicators of NAFLD severity. The current study demonstrated a correlation between sonographic grades of NAFLD and serum levels of TC, HDL-C, TG, and FBS. This aligns with previous research highlighting the intricate interplay between NAFLD and these blood parameters [13-15]. In contrast, ALT, AST, LDL-C, and CRP did not show statistically significant correlations. No correlation was seen with serum ALT and AST.

The results of the present study, when compared to existing literature, show both consistencies and discrepancies. Increasing liver steatosis severity was found to be associated with a statistically significant increase in mean body mass index, serum TG, and FBS in a study published in 2020 [13]. Furthermore, they noted a statistically significant decline in HDL-C with increasing steatosis severity similar to the present study. Contrary to the results of the current study, however, this study [13] also found a correlation between hepatic steatosis with serum ALT and AST, while no statistically significant association was found with TC and LDL-C in our study. Similar results were seen in a study conducted in India showing a statistically significant association of steatosis with raised TC, LDL-C, and very low-density lipoprotein, as well as with low HDL-C [14]. Cuenza et al. in their study reported a statistically significant association of steatosis with TG, ALT, AST, and FBS. However, no statistically significant association was seen with TC, LDL-C, and HDL-C [15]. These discrepancies could be attributed to differences in study population, methodology, and other confounding factors.

The negative correlation observed between CRP levels and NAFLD severity (although not statistically significant) in the present study appears to contradict conventional expectations. A potential explanation for this unexpected finding is the prevalent self-medication with anti-inflammatory drugs and steroids for pain management in Pakistan, particularly among the middle-aged and elderly population. These drugs are known to lower inflammatory indicators like CRP. This may obscure the rise in CRP that would otherwise be associated with worsening NAFLD severity. More research is needed to determine the degree of self-medication in this population and its impact on circulating inflammatory markers.

It is crucial to acknowledge the limitations of the study. The researchers are unable to determine a causal or temporal association between NAFLD grades and blood parameters owing to the cross-sectional nature of the present study. Additionally, the lack of dietary, medication, and lifestyle data limits our ability to comprehensively account for potential confounding variables. Furthermore, the researchers did not exclude patients with potential gallbladder pathologies, as some of these conditions may also occasionally result in hyperechogenicity of the hepatic parenchyma [16].

The current study provides compelling evidence of a correlation between NAFLD grades determined by ultrasound and blood parameters such as TG, TC, LDL-C, and FBS. These findings emphasize the potential clinical utility of blood-based markers in assessing NAFLD severity and its associated metabolic aspects. Additional multicenter longitudinal studies are necessary to validate these results and shed light on the underlying processes causing these relationships. Such research could potentially contribute to improved risk stratification, early diagnosis, and targeted management strategies for individuals with NAFLD.

## Conclusions

This study emphasizes a relationship between NAFLD grade and specific blood parameters. This highlights the importance of employing blood-based indicators to assess the severity and metabolic implications of NAFLD. Additionally, being one of the few studies conducted in Pakistan on this subject, it holds significance since the majority of the population in this study group population avoids alcohol. More long-term multicenter studies could help us better understand the causal mechanisms underlying these associations, which could help us improve risk assessment, early detection, and individualized treatment methods for NAFLD patients. In conclusion, the study found a correlation between hepatic steatosis and blood metabolic markers, implying that controlling these biochemical parameters could potentially improve hepatic steatosis.
